# Disability and in-hospital breastfeeding practices and supports in Ontario, Canada: a population-based study

**DOI:** 10.1016/S2468-2667(22)00310-3

**Published:** 2023-01

**Authors:** Hilary K Brown, Clare Taylor, Simone N Vigod, Cindy-Lee Dennis, Kinwah Fung, Simon Chen, Astrid Guttmann, Susan M Havercamp, Susan L Parish, Joel G Ray, Yona Lunsky

**Affiliations:** Department of Health and Society, University of Toronto Scarborough, Toronto, ON, Canada (H K Brown PhD); Dalla Lana School of Public Health (H K Brown), Department of Psychiatry (S N Vigod MD, C-L Dennis PhD, Y Lunsky PhD) and Department of Pediatrics (A Guttmann MDCM), Temerty Faculty of Medicine and Lawrence S Bloomberg Faculty of Nursing (C-L Dennis), University of Toronto, Toronto, ON, Canada; Women’s College Research Institute, Women’s College Hospital, Toronto, ON, Canada (H K Brown, C Taylor PhD, S N Vigod); ICES, Toronto, ON, Canada (H K Brown, S N Vigod, K Fung MSc, S Chen MPH, A Guttmann, J G Ray MD, Y Lunsky); Li Ka Shing Knowledge Institute, Unity Health Toronto, Toronto, ON, Canada (C-L Dennis, J G Ray); Hospital for Sick Children, Toronto, ON, Canada (A Guttmann); Edwin SH Leong Centre for Healthy Children, Toronto, ON, Canada (A Guttmann); Department of Psychiatry and Behavioral Health, Nisonger Center, Ohio State University, Columbus, OH, USA (S M Havercamp PhD); College of Health Professions, Virginia Commonwealth University, Richmond, VA, USA (S L Parish PhD); Azrieli Adult Neurodevelopmental Centre, Centre for Addiction and Mental Health, Toronto, ON, Canada (Y Lunsky)

## Abstract

**Background:**

Breastfeeding provides infants with nutrients required for optimal growth and development. We aimed to examine breastfeeding practices and supports that promote exclusive breastfeeding during the birth hospital stay among birthing parents with physical disabilities, sensory disabilities, intellectual or developmental disabilities, and multiple disabilities compared with those without a disability.

**Methods:**

This population-based cohort study was done in Ontario, Canada. We accessed and analysed health administrative data from ICES and the Better Outcomes Registry & Network. We included all birthing parents aged 15–49 years who had a singleton livebirth between April 1, 2012, and March 31, 2018. The study outcomes were breastfeeding practices and supports that promoted exclusive breastfeeding during the birth hospital stay, conceptualised based on WHO–UNICEF Baby Friendly Hospital Initiative guidelines. Individuals with a physical disability, sensory disability, intellectual or developmental disability, or two or more (multiple) disabilities, identified using diagnostic algorithms, were compared with individuals without disabilities on the opportunity to initiate breastfeeding, in-hospital breastfeeding, exclusive breastfeeding at hospital discharge, skin-to-skin contact, and provision of breastfeeding assistance. Relative risks (RRs) were estimated using modified Poisson regression.

**Findings:**

Our cohort included 634 111 birthing parents, of whom 54 476 (8·6%) had a physical disability, 19 227 (3·0%) had a sensory disability, 1048 (0·2%) had an intellectual or developmental disability, 4050 (0·6%) had multiple disabilities, and 555 310 (87·6%) had no disability. Individuals with intellectual or developmental disabilities were less likely than those without a disability to have an opportunity to initiate breastfeeding (adjusted RR 0·82, 95% CI 0·76–0·88), any in-hospital breastfeeding (0·85, 0·81–0·88), exclusive breastfeeding at hospital discharge (0·73, 0·67–0·79), skin-to-skin contact (0·90, 0·87–0·94), and breastfeeding assistance (0·85, 0·79–0·91). Those with multiple disabilities were less likely to have an opportunity to initiate breastfeeding (0·93, 0·91–0·96), any in-hospital breastfeeding (0·93, 0·92–0·95), exclusive breastfeeding at hospital discharge (0·90, 0·87–0·93), skin-to-skin contact (0·93, 0·91–0·95), and breastfeeding assistance (0·95, 0·92–0·98). Differences for individuals with a physical or sensory disability only were mostly non-significant.

**Interpretation:**

Our findings show disparities in breastfeeding outcomes between individuals without a disability and individuals with intellectual or developmental disabilities or multiple disabilities, but not individuals with physical or sensory disabilities. There is a need for further research on the factors that contribute to breastfeeding intentions, practices, and supports in people with intellectual or developmental disabilities and multiple disabilities, especially factors that affect breastfeeding decision making.

## Introduction

Breastfeeding is a complex biological and social process with established benefits.^1^ For children, a greater intensity of breastfeeding is protective against otitis media, respiratory tract infections, diarrhoea, malnutrition, and infant mortality; reduces the risk of obesity and type 2 diabetes; and improves cognitive outcomes.^2–5^ Breastfeeding also reduces the risk of breast and ovarian cancers in the birthing parent.^6^ WHO and UNICEF recommend breastfeeding initiation within 1 h of birth and exclusive breastfeeding for 6 months post partum, as part of their Baby Friendly Hospital Initiative.^1^ Skin-to-skin contact immediately after birth promotes breastfeeding initiation and exclusive breastfeeding through improved parental breastfeeding self-efficacy and infant sucking competency.^6^ Therefore, skin-to-skin contact, initiation of breastfeeding, and exclusive breastfeeding in the immediate post-partum period in hospital are clinically important outcomes predictive of long-term breastfeeding.^7^

Despite global efforts to promote skin-to-skin contact, early breastfeeding initiation, and exclusive breastfeeding in hospital, the Baby Friendly Hospital Initiative breastfeeding outcomes remain unmet.^1^ For example, although 81–89% of birthing parents in North America initiate breastfeeding, only half exclusively breastfeed for the first 3 months, dropping to a quarter at 6 months.^1,8^ Young parental age, poverty, lack of social support, smoking, obesity, chronic illness, and perinatal complications are all associated with lower rates of exclusive breastfeeding and shorter breastfeeding duration.^9^ Few studies have examined breastfeeding outcomes in birthing parents with disabilities; this is a substantial gap in evidence, since population-based data in Ontario, Canada show that 1 in 8 pregnancies are to people with a disability,^10^ and people with disabilities have elevated rates of many of the known risk factors for lower rates of breastfeeding.^11–13^ In the few studies on breastfeeding in people with disabilities from the UK and the USA, breastfeeding initiation rates were lower in people with any disability, and in people with intellectual or developmental disabilities, than in those without disabilities.^14–16^ Barriers to breastfeeding in this group might include negative provider attitudes, lack of accessible information, and lack of autonomy.^17,18^ To ensure effective, equitable, and accessible care for all birthing people, further research is needed to inform the development of breastfeeding supports for those with disabilities who wish to breastfeed during the post-partum hospital stay.

We aimed to examine breastfeeding practices and supports that promote exclusive breastfeeding during the birth hospital stay among birthing parents with physical disabilities, sensory disabilities, intellectual or developmental disabilities, and multiple disabilities compared with those without a disability.

## Methods

### Study design and data sources

This population-based cohort study was done in Ontario, Canada. Ontario has 14·7 million residents and approximately 140 000 births each year. We accessed and analysed data from ICES and the Better Outcomes Registry & Network (BORN). ICES is a non-profit research organisation that houses administrative data on the health-care service use of all residents in Ontario, including data on physician visits (Ontario Health Insurance Plan database), emergency department visits (National Ambulatory Care Reporting System), hospital admissions (Canadian Institute for Health Information Discharge Abstract Database; Ontario Mental Health Reporting System), and demographics, such as date of birth, sex, and residential postal code (Registered Persons Database). These datasets were linked using a unique encoded identifier and used to derive the cohort, disability status, and covariates. BORN is a clinical registry that contains obstetric data for all births in Ontario, including data on labour and delivery, post-partum outcomes, infant outcomes from birth to 1 h after birth and from 1 h after birth until discharge, and infant outcomes in the neonatal intensive care unit (NICU). BORN datasets were linked to ICES data with a 93% linkage rate,^19^ and were used to derive breastfeeding outcomes. ICES and BORN data have shown good validity and completeness.^20,21^

ICES is a prescribed entity under Ontario’s Personal Health Information Protection Act (PHIPA). Section 45 of PHIPA authorises ICES to collect personal health information, without consent, for the purpose of analysis or compiling statistical information with respect to the management, evaluation, or monitoring of the allocation of resources to or planning for all or part of the health system. Projects that use data collected by ICES under section 45 of PHIPA, and use no other data, are exempt from research ethics board review. The use of the data in this project was authorised under section 45 and approved by the ICES Privacy and Legal Office.

### Study population

We included all birthing parents aged 15–49 years who had a singleton livebirth between April 1, 2012, and March 31, 2018. We excluded records for which there was a death of the birthing parent or neonate during the birth hospital stay, or where the primary outcome data were missing (appendix pp 1–2). We identified disability status using established algorithms developed to ascertain disability in health administrative data.^22,23^ These algorithms reflect functional limitations, and have been shown to be associated with need for accommodations when accessing health care.^24^ Briefly, we considered a disability to be present if diagnoses were recorded at two or more physician visits, or one or more emergency department visits or hospital admissions, between database inception and conception, as follows: physical disability (congenital anomaly, musculoskeletal disorder, neurological disorder, or permanent injury), sensory disability (hearing loss or vision loss), intellectual or developmental disability (autism spectrum disorder, chromosomal anomaly, fetal alcohol spectrum disorder, or other intellectual disability), or multiple disabilities (two or more of these). The reference group was those without a recorded disability.

### Outcomes

As in our previous research,^25^ the study outcomes were breastfeeding practices and supports that promoted exclusive breastfeeding during the birth hospital stay, conceptualised based on WHO–UNICEF Baby Friendly Hospital Initiative guidelines.^1^ Breastfeeding practices were the opportunity to initiate breastfeeding (ie, to latch) within 2 h of birth, any in-hospital breastfeeding, and provision of exclusive breastfeeding at discharge. Breastfeeding supports were skin-to-skin contact with the birthing parent within 2 h of birth and provision of assistance with breastfeeding within 6 h of birth after initial feeding. We also examined recorded reasons for receiving fluids other than breastmilk, which comprised the following: maternal medical, infant medical, informed parent decision, birth mother not involved in care, and other. Breastfeeding data were collected by hospital staff during the birth hospital stay and recorded directly into the BORN system, or in the medical chart for later abstraction into the BORN system.^19^ Infant feeding data at discharge have been validated against chart re-abstraction, with 76·2% agreement.^21^ The other outcomes have not been validated, but have been used in our previous research.^25^

### Covariates

We measured confounders and potential pathway variables (appendix pp 3–4). Confounders were factors associated with disability and the study outcomes, but not on the causal pathway between them, including age, parity, neighbourhood income quintile, rural residence, and comorbidities.^11–13^ Neighbourhood income quintile was measured by linking postal codes with census-area-level income data. Rural residence was measured using the Rurality Index of Ontario,^26^ which uses ten indicators to classify areas as rural or urban. Comorbidities were chronic conditions, mental ill health, and substance use disorders in the 2 years before conception. We used the Johns Hopkins Adjusted Clinical Groups System version 10 collapsed ambulatory diagnostic groups to identify chronic conditions.^27^ Chronic conditions were classified as stable or unstable, with the latter defined on the basis of the likelihood of complications and need for resources such as specialist care.^27^ Mental ill health (ie, psychosis, mood or anxiety disorder, or other) and substance use disorders were based on at least two physician visits, at least one emergency department visit, or hospital admission.

Pathway variables were factors that could explain the relationship between disability and the study outcomes, and included smoking in pregnancy, overweight or obesity, prenatal care characteristics, breastfeeding intentions, and factors that could signal parental–infant separations. Smoking in pregnancy was defined as any cigarette smoking at the first prenatal visit or at birth. Overweight or obesity was measured using pre-pregnancy BMI. Prenatal care characteristics were type of prenatal care provider (ie, family physician, obstetrician, midwife, shared care, or none), number of prenatal care visits (ie, fewer than recommended, ten or less; recommended, 11–14; more than recommended, 15 or more);^28^ and prenatal class attendance. We collected self-reported intention to breastfeed during prenatal care or at birth. Finally, we measured factors that could signal separation of the birthing parent and infant during the birth hospital stay, inclusing caesarean delivery, severe maternal morbidity, preterm birth before 37 weeks, NICU admission, and infant discharge to social services.

### Statistical analysis

We described the characteristics of birthing parents with disabilities using frequencies and percentages, with comparisons to those without disabilities quantified using standardised differences.^29^ Standardised differences are appropriate for large cohorts since, unlike p values, they do not depend on sample size; differences greater than 0·10 show meaningful imbalances.^29^

We used modified Poisson regression, with generalised estimating equations to account for multiple births to the same person during the study period,^30^ to calculate relative risks (RRs) and 95% CIs for breastfeeding practices and supports in each group of people with disabilities compared with those with no disability. Covariates were added to the models in three steps, as follows: first, we controlled for age; second, we controlled for age and the remaining prespecified confounders, parity, neighbourhood income quintile, rural residence, chronic conditions, mental illness, and substance use disorders (main model); and third, we controlled for the confounders in the second step and the possible pathway variables, smoking in pregnancy, overweight or obesity, prenatal care provider type, number of prenatal care visits, and prenatal class attendance. This third analysis tested the effect of the pathway variables on the results of the main models.

In additional analyses, we restricted the models to low-risk pregnancies (ie, full-term vaginal births to individuals without severe maternal morbidity, in which there was no NICU admission or newborn discharge to social services) and to those who intended to breastfeed. We also reran the models by disability subtype, within each of the four categories of disability. Finally, we investigated the reasons for not exclusively breastfeeding during the birth hospital stay.

Covariate information was missing for a small proportion of the cohort, with the highest level of missingness for BMI, at 14·6% (n=92 580). Therefore, we used multiple imputation with a chained equation approach; in a subset of those with complete covariate information, 15 imputed datasets were created and combined using Rubin’s rule.^31^ SAS 9.4 was used for all analyses.

### Role of the funding source

The funders of the study had no role in the study design, data collection, data analysis, data interpretation, or writing of the report.

## Results

Our cohort included 634 111 birthing parents, of whom 54 476 (8·6%) had a physical disability, 19 227 (3·0%) had a sensory disability, 1048 (0·2%) had an intellectual or developmental disability, 4050 (0·6%) had multiple disabilities, and 555 310 (87·6%) had no disability (table 1). Compared with people without a disability, those with intellectual or developmental disabilities tended to be younger and, along with people with multiple disabilities, were more likely to live in low-income neighbourhoods. People with multiple disa bilities were more likely to have any type of chronic condition, and those with physical disabilities and intellectual or developmental disabilities were more likely to have unstable chronic conditions. People with disabilities were more likely to have mental ill health, and those with physical disabilities, intellectual or developmental disabilities, and multiple disabilities were more likely to have a substance use disorder. People with disabilities were more likely to smoke during pregnancy, and those with physical, sensory, and multiple disabilities were more likely to be overweight or obese. People with physical, intellectual or developmental, and multiple disabilities were less likely to have an intention to breastfeed. Those with intellectual or developmental disabilities or multiple disabilities were more likely to have severe maternal morbidity, preterm birth, infant NICU admission, and infant discharge to social services, and those with multiple disabilities were more likely to have a caesarean section, compared with people without disabilities (table 1).

305 882 (55·1%) of 555 310 people without a disability had the opportunity to initiate breastfeeding within 2 h of birth, compared with 29 405 (54·0%) of 54 476 individuals with a physical disability (adjusted RR 0·99, 0·98–1·00), 10 499 (54·6%) of 19 227 individuals with a sensory disability (1·00, 0·98–1·01), 447 (42·7%) of 1048 individuals with an intellectual or developmental disability (0·82, 0·76–0·88), and 2027 (50·0%) of 4050 individuals with multiple disabilities (0·93, 0·91–0·96; table 2). 482 702 (86·9%) of 555 310 people without a disability had any in-hospital breastfeeding, compared with 45 881 (84·2%) of 54 476 individuals with a physical disability (0·98, 0·97–0·99), 16 279 (84·7%) of 19 227 individuals with a sensory disability (0·98, 0·97–0·99), 731 (69·8%) of 1048 individuals with an intellectual or developmental disability (0·85, 0·81–0·88), and 3187 (78·7%) of 4050 individuals with multiple disabilities (0·93, 0·92–0·95; table 2). 327 981 (59·1%) of 555 310 people without a disability were exclusively breastfeeding at hospital discharge, compared with 31 596 (58·0%) of 54 476 individuals with a physical disability (1·00, 0·99–1·00), 11 146 (58·0%) of 19 227 individuals with a sensory disability (0·99, 0·98–1·00), 414 (39·5%) of 1048 individuals with an intellectual or developmental disability (0·73, 0·67–0·79), and 2065 (51·0%) of 4050 individuals with multiple disabilities (0·90, 0·87–0·93; table 2). Observed associations were only slightly reduced after further adjustment for possible pathway variables, with the exception of any in-hospital breastfeeding, which no longer showed a significant difference for people with sensory disabilities (table 2).

430 762 (77·6%) of 555 310 people without a disability had skin-to-skin contact with their infant within 2 h of birth, compared with 41 546 (76·3%) of 54 476 individuals with a physical disability (adjusted RR 0·99, 95% CI 0·98–0·99), 14 600 (75·9%) of 19 227 individuals with a sensory disability (0·98, 0·97–0·99), 720 (68·7%) of 1048 individuals with an intellectual or developmental disability (0·90, 0·87–0·94), and 2886 (71·3%) of 4050 individuals with multiple disabilities (0·93, 0·91–0·95; table 2). 297 278 (53·5%) of 555 310 people without a disability had assistance provided with breastfeeding within 6 h of birth after the initial feeding, compared with 28 672 (52·6%) of 54 476 individuals with a physical disability (0·99, 0·98–1·00), 10 196 (53·0%) of 19 227 individuals with a sensory disability (1·00, 0·98–1·01), 457 (43·6%) of 1048 individuals with an intellectual or developmental disability (0·85, 0·79–0·91), and 1996 (49·3%) of 4050 individuals with multiple disabilities (0·95, 0·92–0·98; table 2). Observed associations were reduced after further adjustment for possible pathway variables, with receipt of skin-to-skin contact no longer showing a statistically significant difference for people with physical and sensory disabilities, and provision of assistance with breastfeeding no longer showing a statistically significant difference for those with multiple disabilities (table 2).

After we restricted the cohort to low-risk births (table 3) and to people who intended to breastfeed (table 4), associations were notably attenuated. Only our findings for exclusive breastfeeding at discharge remained both statistically significant and with similar magnitude to the non-restricted results.

Analyses by subtype of disability within each of the four disability categories revealed that findings for individuals with multiple disabilities, although mostly statistically significant across outcomes, showed the largest disparities in people who had an intellectual or developmental disability plus a physical or sensory disability (or both; appendix p 4).

Reasons for receiving fluids other than breastmilk were mostly similar across groups, but people with intellectual or developmental disabilities were less likely than those without disabilities to have infant medical reasons listed, and were more likely to have birth mother not involved in care recorded as the reason, whereas those with multiple disabilities were more likely to have maternal medical reasons or birth mother not involved in care recorded compared with those without disabilities (appendix p 8).

## Discussion

In this large, population-based study, we found that more could be done to increase levels of breastfeeding for all Ontario birthing parents who wish to breastfeed, and particularly those with intellectual or developmental disabilities or multiple disabilities, who had lower rates of breastfeeding initiation within 2 h of birth, any in-hospital breastfeeding, exclusive breastfeeding at hospital discharge, skin-to-skin contact within 2 h of birth, and receipt of professional breastfeeding support within 6 h of birth compared with those without disabilities. Small disparities in exclusive breastfeeding at discharge persisted after restricting the cohort to low-risk births and to people who intended to breastfeed, whereas disparities for other breastfeeding practices and supports were largely attenuated after these restrictions were applied. Associations were also weakened after adjustment for smoking, BMI, and prenatal care access. Our data indicate a need for a better understanding of factors that affect breastfeeding intentions, practices, and supports in people with intellectual or developmental disabilities and multiple disabilities, to inform the development of early and accessible breastfeeding resources.

To our knowledge, only three quantitative studies have examined breastfeeding in people with disabilities. A UK survey found that 70% of people with disabilities initiated breastfeeding within the first few days post partum, compared with 79% of those without disabilities.^14^ In the USA, data from the Rhode Island Pregnancy Risk Assessment Monitoring System showed 70% of people with disabilities who recently gave birth reported ever breastfeeding and 45% were currently breastfeeding, compared with 75% and 53% of those without disabilities, respectively.^15^ Also in the USA, data from the Massachusetts Pregnancy to Early Life Longitudinal database showed breastfeeding at discharge was reported by 49% of people with intellectual or developmental disabilities compared with 74% of those with diabetes and 77% of those with neither condition, an intellectual or developmental disability, or diabetes.^16^ Our study adds to this body of literature by being the first, to our knowledge, to address WHO–UNICEF Baby Friendly Hospital Initiative indicators in people with disabilities,^1^ including breastfeeding initiation, exclusive breastfeeding at discharge, and skin-to-skin contact—all of which predict exclusive breastfeeding at 6 months.^7^ To our knowledge, our study is also the first to examine gaps in receipt of professional breastfeeding supports between people with disabilities and people without disabilities and is one of the first cross-disability studies, showing disparities for people with intellectual or developmental disabilities and multiple disabilities, but not for those with physical or sensory disabilities.

There were few differences in breastfeeding practices and supports for people with physical and sensory disabilities compared with those without disabilities. However, the reasons for the disparities between people with intellectual or developmental disabilities or multiple disabilities and people without disabilities warrant further exploration. Only disparities in exclusive breastfeeding at hospital discharge persisted after restricting the cohort to people who intended to breastfeed and low-risk pregnancies, suggesting that early decisions about breastfeeding and birthing parent–neonate separations are important drivers of disparities in people with disabilities. Associations between disability status and the study outcomes were also weakened after adding pathway variables such as smoking and overweight or obesity—which are known predictors of breastfeeding^9^—to the models. Factors that were not investigated in our study might also be important, including medication use, pain and fatigue, understanding of the benefits of breastfeeding, and comfort with breastfeeding. Structural barriers might also have a role. In qualitative studies, for example, people with disabilities report that health-care providers do not have information on how breastfeeding interacts with disability or adaptive breastfeeding techniques,^17^ and providers sometimes discourage people with disabilities from breastfeeding, even when it is not contraindicated.^18^ For those with intellectual or developmental disabilities in particular, providers might also assume that parents lack ability (eg, to understand let-down sensation, infant sucking, or the amount of breastfeeding required). Health-care provider training and attitudes might therefore be important contributors that warrant further investigation.

Our study has several limitations. Measurement of disability from health administrative data captures diagnosis-based impairments, but not activity limitations or participation restrictions. Disability status might have been misclassified if diagnoses were not recorded or if people did not access care for their disability;^24^ such misclassification might result in conservative risk estimates. We conceptualised mental ill health as a comorbidity rather than part of our definition of disability; however, other studies have found an association between mental ill health and barriers to breastfeeding.^25^ Breastfeeding outcomes were restricted to the birth hospital stay, and might not reflect practices and supports thereafter. However, breastfeeding initiation, exclusive breastfeeding, and skin-to-skin contact in hospital are predictors of later breastfeeding,^7^ are critical for receipt of nutrient-rich colostrum, and might be important for parent–infant bonding. Some breastfeeding data were missing, but there were few differences in baseline characteristics when comparing those who were or were not excluded. Although many of the breastfeeding variables in this study reflect quality of care indicators tracked by the WHO and UNICEF, there were some differences (eg, breastfeeding initiation was measured within 2 h of birth, not 1 h as in Baby Friendly Hospital Initiative indicators).^1^ The categories of reasons for receiving fluids other than breastmilk were broad (eg, “birth mother not involved in care” might reflect various issues). With respect to breastfeeding intentions, it is not known if individual intentions changed during pregnancy, and how the final intention value was chosen. We did not have data on individual factors such as relationship status and other supports, medication or other substance use in pregnancy, and pain. We were also constrained in terms of ascertainment of demographic data, including gender identity and race and ethnicity, which might be associated with other structural barriers to care. Finally, we did not have data on system-level factors, such as health-care provider attitudes.

In conclusion, our findings have important implications. Although the absence of significant differences between people with physical and sensory disabilities and those without disabilities was a positive finding, more could be done to increase levels of breastfeeding for Ontario birthing parents who wish to breastfeed, and particularly people with intellectual or developmental disabilities or multiple disabilities. There is a need for further research on the factors that contribute to breastfeeding intentions, practices, and supports in people with intellectual or developmental disabilities and multiple disabilities, especially factors that affect breastfeeding decision making. Such research might inform training for health-care providers—including nurses, lactation consultants, and paediatricians—on disability, accessible communication and learning needs, adapted breastfeeding techniques and supports for high-risk births, and the intersection between disability and other structural barriers to breastfeeding. Such research could also inform preconception and prenatal health promotion and education efforts, supporting informed decision making in people with intellectual or developmental disabilities or multiple disabilities, including early and accessible information on the benefits of breastfeeding,^2–6^ and tailored supports to increase breastfeeding self-efficacy and address barriers. All these efforts should be done in partnership with disability advocacy and support organisations to ensure that they are developed based on the needs and preferences of people with disabilities.

## Supplementary Material

1

## Figures and Tables

**Table 1:** Baseline characteristics of those aged 15–49 years with a physical, sensory, or intellectual or developmental disability, or multiple disabilities, and those without a recognised disability

	All (n=634 111)	Physical disability only (n=54 476)	Sensory disability only (n=19 227)	Intellectual or developmental disability only (n=1048)	Multiple disabilities (n=4050)	No disability (n=555 310)
Age, years						
15–24	22 218 (3·5%)	1959 (3·6%)	880 (4·6%)	169 (16·1%)*	182 (4·5%)	19 028 (3·4%)
25–34	494 845 (78·0%)	41 414 (76·0%)	15 051 (78·3%)	771 (73·6%)*	3134 (77·4%)	434 475 (78·2%)
35–49	117 048 (18·5%)	11 103 (20·4%)	3296 (17·1%)	108 (10·3%)*	734 (18·1%)	101 807 (18·3%)
Multiparous	364 550 (57·5%)	31 867 (58·5%)	10 576 (55·0%)	573 (54·7%)	2317 (57·2%)	319 217 (57·5%)
Neighbourhood income quintile						
1 (lowest)	140 309 (22·1%)	11 802 (21·7%)	4295 (22·3%)	415 (39·6%)*	1067 (26·3%)*	122 730 (22·1%)
2	127 459 (20·1%)	10 842 (19·9%)	3937 (20·5%)	219 (20·9%)	830 (20·5%)	111 631 (20·1%)
3	129 733 (20·5%)	11 140 (20·4%)	3875 (20·2%)	179 (17·1%)	806 (19·9%)	113 733 (20·5%)
4	131 829 (20·8%)	11 469 (21·1%)	4098 (21·3%)	116 (11·1%)*	743 (18·3%)	115 403 (20·8%)
5 (highest)	102 657 (16·2%)	9010 (16·5%)	2956 (15·4%)	108 (10·3%)*	587 (14·5%)	89 996 (16·2%)
Missing	2124 (0·3%)	213 (0·4%)	66 (0·3%)	11 (1·0%)	17 (0·4%)	1817 (0·3%)
Rural region of residence	29 773 (4·7%)	3299 (6·1%)	927 (4·8%)	49 (4·7%)	250 (6·2%)*	25 248 (4·5%)
Stable chronic conditions	142 979 (22·5%)	14 086 (25·9%)	4827 (25·1%)	258 (24·6%)	1238 (30·6%)*	122 570 (22·1%)
Unstable chronic conditions	80 456 (12·7%)	9073 (16·7%)*	2859 (14·9%)	177 (16·9%)*	898 (22·2%)*	67 449 (12·1%)
Mental ill health	237 819 (37·5%)	29 172 (53·6%)*	9040 (47·0%)*	804 (76·7%)*	2574 (63·6%)*	196 229 (35·3%)
Substance use disorder	26 613 (4·2%)	3963 (7·3%)*	964 (5·0%)	218 (20·8%)*	467 (11·5%)*	21 001 (3·8%)
Prenatal smoking	69 235 (10·9%)	8751 (16·1%)*	2662 (13·8%)*	354 (33·8%)*	839 (20·7%)*	56 629 (10·2%)
Pre-pregnancy overweight or obesity	250 732 (39·5%)	25 001 (45·9%)*	8371 (43·5%)*	410 (39·1%)	1896 (46·8%)*	215 054 (38·7%)
Type of prenatal care provider						
Family physician	96 606 (15·2%)	8488 (15·6%)	2984 (15·5%)	184 (17·6%)	591 (14·6%)	84 359 (15·2%)
Obstetrician	223 894 (35·3%)	18 907 (34·7%)	6810 (35·4%)	366 (34·9%)	1408 (34·8%)	196 403 (35·4%)
Midwife	7073 (1·1%)	604 (1·1%)	235 (1·2%)	14 (1·3%)	36 (0·9%)	6184 (1·1%)
Shared care	260 497 (41·1%)	22 897 (42·0%)	7843 (40·8%)	436 (41·6%)	1797 (44·4%)	227 524 (41·0%)
None	46 041 (7·3%)	3580 (6·6%)	1355 (7·0%)	48 (4·6%)*	218 (5·4%)	40 840 (7·4%)
Number of prenatal care visits						
≤10	144 617 (22·8%)	12 256 (22·5%)	4375 (22·8%)	260 (24·8%)	867 (21·4%)	126 859 (22·8%)
11–14	171 795 (27·1%)	14 135 (25·9%)	4975 (25·9%)	247 (23·6%)	1009 (24·9%)	151 429 (27·3%)
≥15	317 699 (50·1%)	28 085 (51·6%)	9877 (51·4%)	541 (51·6%)	2174 (53·7%)	277 022 (49·9%)
Prenatal class attendance	142 879 (22·5%)	12 120 (22·2%)	4719 (24·5%)	252 (24·0%)	923 (22·8%)	124 865 (22·5%)
Intention to breastfeed	565 051 (89·1%)	47 412 (87·0%)*	16 933 (88·1%)	764 (72·9%)*	3362 (83·0%)*	496 580 (89·4%)
Caesarean delivery	171 642 (27·1%)	16 189 (29·7%)	5404 (28·1%)	285 (27·2%)	1313 (32·4%)*	148 451 (26·7%)
Severe maternal morbidity	12 439 (2·0%)	1347 (2·5%)	424 (2·2%)	37 (3·5%)*	152 (3·8%)*	10 479 (1·9%)
Preterm birth before 37 weeks	37 711 (5·9%)	3848 (7·1%)	1321 (6·9%)	95 (9·1%)*	377 (9·3%)*	32 070 (5·8%)
Neonatal intensive care unit admission	73 617 (11·6%)	7622 (14·0%)	2440 (12·7%)	233 (22·2%)*	729 (18·0%)*	62 593 (11·3%)
Infant discharge to social services	2499 (0·4%)	367 (0·7%)	118 (0·6%)	91 (8·7%)*	97 (2·4%)*	1826 (0·3%)

Data are n (%).

* Standardised difference >0·10, comparing people within each respective disability group to those without a disability.

**Table 2:** Breastfeeding practices and receipt of supports that promote exclusive breastfeeding during the birth hospital stay, in people with a disability and in those without a recognised disability

	Individuals with outcome	Unadjusted RR (95% CI)	Adjusted RR (95% CI)*	Adjusted RR (95% CI)^†^	Adjusted RR (95% CI)^‡^
**Opportunity to initiate breastfeeding within 2 h of birth**					
No disability (n=555 310)	305 882 (55·1%)	1 (ref)	1 (ref)	1 (ref)	1 (ref)
Physical disability only (n=54 476)	29 405 (54·0%)	0·98 (0·97–0·99)	0·98 (0·97–0·99)	0·99 (0·98–1·00)	1·00 (0·99–1·01)
Sensory disability only (n=19 227)	10 499 (54·6%)	0·99 (0·98–1·01)	0·99 (0·98–1·01)	1·00 (0·98–1·01)	1·00 (0·98–1·02)
Intellectual or developmental disability only (n=1048)	447 (42·7%)	0·77 (0·72–0·83)	0·79 (0·73–0·85)	0·82 (0·76–0·88)	0·84 (0·76–0·92)
Multiple disabilities (n=4050)	2027 (50·0%)	0·91 (0·88–0·94)	0·91 (0·89–0·94)	0·93 (0·91–0·96)	0·95 (0·91–0·99)
**Any in-hospital breastfeeding**					
No disability (n=555 310)	482 702 (86·9%)	1 (ref)	1 (ref)	1 (ref)	1 (ref)
Physical disability only (n=54 476)	45 881 (84·2%)	0·97 (0·96–0·97)	0·97 (0·96–0·97)	0·98 (0·97–0·98)	0·99 (0·98–0·99)
Sensory disability only (n=19 227)	16 279 (84·7%)	0·97 (0·97–0·98)	0·97 (0·97–0·98)	0·98 (0·97–0·99)	0·98 (0·97–1·00)
Intellectual or developmental disability only (n=1048)	731 (69·8%)	0·81 (0·77–0·84)	0·82 (0·79–0·86)	0·85 (0·81–0·88)	0·86 (0·80–0·93)
Multiple disabilities (n=4050)	3187 (78·7%)	0·91 (0·90–0·93)	0·91 (0·90–0·93)	0·93 (0·92–0·95)	0·94 (0·91–0·98)
**Exclusive breastfeeding at hospital discharge**					
No disability (n=555 310)	327 981 (59·1%)	1 (ref)	1 (ref)	1 (ref)	1 (ref)
Physical disability only (n=54 476)	31 596 (58·0%)	0·98 (0·97–0·99)	0·98 (0·98–0·99)	1·00 (0·99–1·00)	1·01 (1·00–1·02)
Sensory disability only (n=19 227)	11 146 (58·0%)	0·98 (0·97–0·99)	0·98 (0·97–0·99)	0·99 (0·98–1·00)	1·00 (0·98–1·02)
Intellectual or developmental disability only (n=1048)	414 (39·5%)	0·69 (0·63–0·74)	0·69 (0·63–0·74)	0·73 (0·67–0·79)	0·75 (0·68–0·83)
Multiple disabilities (n=4050)	2065 (51·0%)	0·87 (0·84–0·90)	0·87 (0·84–0·90)	0·90 (0·87–0·93)	0·93 (0·89–0·97)
**Skin-to-skin contact with the birthing parent within 2 h of birth**					
No disability (n=555 310)	430 762 (77·6%)	1 (ref)	1 (ref)	1 (ref)	1 (ref)
Physical disability only (n=54 476)	41 546 (76·3%)	0·98 (0·98–0·99)	0·98 (0·98–0·99)	0·99 (0·98–0·99)	0·99 (0·98–1·00)
Sensory disability only (n=19 227)	14 600 (75·9%)	0·98 (0·97–0·99)	0·98 (0·97–0·99)	0·98 (0·97–0·99)	0·98 (0·97–1·00)
Intellectual or developmental disability only (n=1048)	720 (68·7%)	0·89 (0·85–0·93)	0·89 (0·85–0·92)	0·90 (0·87–0·94)	0·91 (0·84–0·98)
Multiple disabilities (n=4050)	2886 (71·3%)	0·92 (0·90–0·94)	0·92 (0·90–0·94)	0·93 (0·91–0·95)	0·94 (0·90–0·97)
**Provision of assistance with breastfeeding within 6 h of birth after the initial feeding**					
No disability (n=555 310)	297 278 (53·5%)	1 (ref)	1 (ref)	1 (ref)	1 (ref)
Physical disability only (n=54 476)	28 672 (52·6%)	0·99 (0·98–1·00)	0·99 (0·98–1·00)	0·99 (0·98–1·00)	1·00 (0·99–1·01)
Sensory disability only (n=19 227)	10 196 (53·0%)	0·99 (0·98–1·01)	0·99 (0·98–1·01)	1·00 (0·98–1·01)	1·00 (0·98–1·02)
Intellectual or developmental disability only (n=1048)	457 (43·6%)	0·82 (0·77–0·88)	0·82 (0·76–0·88)	0·85 (0·79–0·91)	0·87 (0·79–0·95)
Multiple disabilities (n=4050)	1996 (49·3%)	0·93 (0·90–0·96)	0·93 (0·90–0·96)	0·95 (0·92–0·98)	0·96 (0·92–1·01)

Data are n (%), unless otherwise indicated. RR=relative risk.

* Adjusted for age.

† Adjusted for age, parity, neighbourhood income quintile, rural residence, stable and unstable chronic conditions, mental ill health, and substance use disorders.

‡ Adjusted for age, parity, neighbourhood income quintile, rural residence, stable and unstable chronic conditions, mental ill health, substance use disorders, smoking, overweight or obesity, prenatal care provider type, number of prenatal care visits, and prenatal class attendance.

**Table 3:** Breastfeeding practices and receipt of supports that promote exclusive breastfeeding during the birth hospital stay, in people with a disability and in those without a recognised disability, restricted to full-term vaginal births to people without severe maternal morbidity, in which there was no neonatal intensive care unit admission or infant discharge to social services

	Individuals with outcome	Unadjusted RR (95% CI)	Adjusted RR (95% CI)*	Adjusted RR (95% CI)^†^	Adjusted RR (95% CI)^‡^
**Opportunity to initiate breastfeeding within 2 h of birth**					
No disability (n=356 915)	215 798 (60·5%)	1 (ref)	1 (ref)	1 (ref)	1 (ref)
Physical disability only (n=32 567)	19 823 (60·9%)	1·01 (1·00–1·02)	1·01 (1·00–1·02)	1·01 (1·00–1·02)	1·01 (1·00–1·03)
Sensory disability only (n=11 944)	7267 (60·8%)	1·01 (0·99–1·02)	1·01 (0·99–1·02)	1·01 (0·99–1·03)	1·01 (0·99–1·04)
Intellectual or developmental disability only (n=557)	303 (54·4%)	0·90 (0·83–0·97)	0·91 (0·84–0·99)	0·93 (0·86–1·00)	0·95 (0·85–1·06)
Multiple disabilities (n=2198)	1323 (60·2%)	1·00 (0·96–1·03)	1·00 (0·96–1·03)	1·00 (0·97–1·04)	1·02 (0·96–1·07)
**Any in-hospital breastfeeding**					
No disability (n=356 915)	317 060 (88·8%)	1 (ref)	1 (ref)	1 (ref)	1 (ref)
Physical disability only (n=32 567)	28 385 (87·2%)	0·98 (0·98–0·99)	0·98 (0·98–0·99)	0·99 (0·98–0·99)	0·99 (0·98–1·00)
Sensory disability only (n=11 944)	10 425 (87·3%)	0·98 (0·97–0·99)	0·98 (0·98–0·99)	0·99 (0·98–0·99)	0·99 (0·97–1·01)
Intellectual or developmental disability only (n=557)	436 (78·3%)	0·88 (0·84–0·92)	0·90 (0·86–0·94)	0·92 (0·88–0·96)	0·93 (0·85–1·03)
Multiple disabilities (n=2198)	1830 (83·3%)	0·94 (0·92–0·96)	0·95 (0·93–0·96)	0·96 (0·94–0·98)	0·97 (0·92–1·01)
**Exclusive breastfeeding at hospital discharge**					
No disability (n=356 915)	241 448 (67·6%)	1 (ref)	1 (ref)	1 (ref)	1 (ref)
Physical disability only (n=32 567)	22 156 (68·0%)	1·01 (1·00–1·01)	1·01 (1·00–1·01)	1·01 (1·00–1·02)	1·02 (1·01–1·03)
Sensory disability only (n=11 944)	8078 (67·6%)	1·00 (0·99–1·01)	1·00 (0·99–1·02)	1·01 (1·00–1·02)	1·01 (0·99–1·03)
Intellectual or developmental disability only (n=557)	293 (52·6%)	0·78 (0·72–0·85)	0·80 (0·74–0·87)	0·83 (0·77–0·90)	0·85 (0·76–0·96)
Multiple disabilities (n=2198)	1391 (63·3%)	0·94 (0·91–0·97)	0·94 (0·91–0·98)	0·96 (0·93–0·99)	0·98 (0·93–1·03)
**Skin-to-skin contact with the birthing parent within 2 h of birth**					
No disability (n=356 915)	307 380 (86·1%)	1 (ref)	1 (ref)	1 (ref)	1 (ref)
Physical disability only (n=32 567)	28 220 (86·7%)	1·01 (1·00–1·01)	1·01 (1·00–1·01)	1·01 (1·00–1·01)	1·01 (0·99–1·02)
Sensory disability only (n=11 944)	10 196 (85·4%)	0·99 (0·98–1·00)	0·99 (0·98–1·00)	0·99 (0·98–1·00)	0·99 (0·97–1·01)
Intellectual or developmental disability only (n=557)	484 (86·9%)	1·01 (0·98–1·04)	1·01 (0·98–1·05)	1·01 (0·98–1·04)	1·01 (0·93–1·11)
Multiple disabilities (n=2198)	1848 (84·1%)	0·98 (0·96–1·00)	0·98 (0·96–0·99)	0·97 (0·96–0·99)	0·98 (0·93–1·02)
**Provision of assistance with breastfeeding within 6 h of birth after the initial feeding**					
No disability (n=356 915)	203 566 (57·0%)	1 (ref)	1 (ref)	1 (ref)	1 (ref)
Physical disability only (n=32 567)	18 588 (57·1%)	1·00 (0·99–1·01)	1·00 (0·99–1·01)	1·00 (0·99–1·01)	1·00 (0·99–1·02)
Sensory disability only (n=11 944)	6771 (56·7%)	0·99 (0·98–1·01)	0·99 (0·98–1·01)	1·00 (0·98–1·01)	1·00 (0·97–1·02)
Intellectual or developmental disability only (n=557)	297 (53·3%)	0·93 (0·86–1·00)	0·92 (0·85–1·00)	0·94 (0·87–1·02)	0·96 (0·85–1·08)
Multiple disabilities (n=2198)	1208 (55·0%)	0·96 (0·92–1·00)	0·96 (0·92–1·00)	0·96 (0·93–1·00)	0·98 (0·92–1·04)

Data are n (%), unless otherwise indicated. RR=relative risk.

* Adjusted for age.

† Adjusted for age, parity, neighbourhood income quintile, rural residence, stable and unstable chronic conditions, mental ill health, and substance use disorders.

‡ Adjusted for age, parity, neighbourhood income quintile, rural residence, stable and unstable chronic conditions, mental ill health, substance use disorders, smoking, overweight or obesity, prenatal care provider type, number of prenatal care visits, and prenatal class attendance.

**Table 4:** Breastfeeding practices and receipt of supports that promote exclusive breastfeeding during the birth hospital stay, in people with a disability and in those without a recognised disability, restricted to women who reported an intention to breastfeed

	Individuals with outcome	Unadjusted RR (95% CI)	Adjusted RR (95% CI)*	Adjusted RR (95% CI)^†^	Adjusted RR (95% CI)^‡^
**Opportunity to initiate breastfeeding within 2 h of birth**					
No disability (n=496 580)	294 807 (59·4%)	1 (ref)	1 (ref)	1 (ref)	1 (ref)
Physical disability only (n=47 412)	28 299 (59·7%)	1·01 (1·00–1·01)	1·01 (1·00–1·01)	1·01 (1·00–1·02)	1·01 (1·00–1·02)
Sensory disability only (n=16 933)	10 104 (59·7%)	1·01 (0·99–1·02)	1·00 (0·99–1·02)	1·01 (0·99–1·02)	1·01 (0·99–1·03)
Intellectual or developmental disability only (n=764)	419 (54·8%)	0·92 (0·87–0·99)	0·93 (0·87–0·99)	0·94 (0·88–1·00)	0·95 (0·86–1·05)
Multiple disabilities (n=3362)	1932 (57·5%)	0·97 (0·94–1·00)	0·97 (0·94–1·00)	0·98 (0·95–1·01)	0·99 (0·94–1·03)
**Any in-hospital breastfeeding**					
No disability (n=496 580)	462 361 (93·1%)	1 (ref)	1 (ref)	1 (ref)	1 (ref)
Physical disability only (n=47 412)	43 912 (92·6%)	0·99 (0·99–1·00)	0·99 (0·99–1·00)	1·00 (0·99–1·00)	1·00 (0·99–1·01)
Sensory disability only (n=16 933)	15 603 (92·1%)	0·99 (0·98–0·99)	0·99 (0·99–0·99)	0·99 (0·99–1·00)	0·99 (0·98–1·01)
Intellectual or developmental disability only (n=764)	659 (86·3%)	0·93 (0·90–0·95)	0·93 (0·91–0·96)	0·94 (0·92–0·97)	0·95 (0·88–1·02)
Multiple disabilities (n=3362)	3017 (89·7%)	0·96 (0·95–0·98)	0·97 (0·95–0·98)	0·97 (0·96–0·98)	0·98 (0·94–1·01)
**Exclusive breastfeeding at hospital discharge**					
No disability (n=496 580)	317 481 (63·9%)	1 (ref)	1 (ref)	1 (ref)	1 (ref)
Physical disability only (n=47 412)	30 598 (64·5%)	1·01 (1·00–1·02)	1·01 (1·00–1·02)	1·02 (1·01–1·02)	1·03 (1·01–1·04)
Sensory disability only (n=16 933)	10 810 (63·8%)	1·00 (0·99–1·01)	1·00 (0·98–1·01)	1·00 (0·99–1·01)	1·01 (0·99–1·02)
Intellectual or developmental disability only (n=764)	382 (50·0%)	0·79 (0·74–0·85)	0·79 (0·73–0·85)	0·81 (0·76–0·88)	0·82 (0·74–0·91)
Multiple disabilities (n=3362)	1986 (59·1%)	0·92 (0·90–0·95)	0·92 (0·90–0·95)	0·94 (0·92–0·97)	0·96 (0·92–1·01)
**Skin-to-skin contact with the birthing parent within 2 h of birth**					
No disability (n=496 580)	392 395 (79·0%)	1 (ref)	1 (ref)	1 (ref)	1 (ref)
Physical disability only (n=47 412)	37 198 (78·5%)	0·99 (0·99–1·00)	0·99 (0·99–1·00)	1·00 (0·99–1·00)	1·00 (0·99–1·01)
Sensory disability only (n=16 933)	13 197 (77·9%)	0·99 (0·98–0·99)	0·98 (0·98–0·99)	0·99 (0·98–1·00)	0·99 (0·97–1·00)
Intellectual or developmental disability only (n=764)	583 (76·3%)	0·97 (0·93–1·01)	0·96 (0·92–1·00)	0·97 (0·93–1·01)	0·97 (0·90–1·06)
Multiple disabilities (n=3362)	2516 (74·8%)	0·95 (0·93–0·97)	0·95 (0·93–0·97)	0·95 (0·94–0·97)	0·96 (0·92–1·00)
**Provision of assistance with breastfeeding within 6 h of birth after the initial feeding**					
No disability (n=496 580)	286 724 (57·7%)	1·00 (Referent)	1·00 (Referent)	1·00 (Referent)	1·00 (Referent)
Physical disability only (n=47 412)	27 623 (58·3%)	1·01 (1·00–1·02)	1·01 (1·00–1·02)	1·01 (1·00–1·02)	1·01 (1·00–1·03)
Sensory disability only (n=16 933)	9831 (58·1%)	1·01 (1·00–1·02)	1·01 (0·99–1·02)	1·01 (1·00–1·02)	1·01 (0·99–1·03)
Intellectual or developmental disability only (n=764)	416 (54·5%)	0·95 (0·90–1·02)	0·94 (0·88–1·00)	0·95 (0·89–1·02)	0·96 (0·87–1·06)
Multiple disabilities (n=3362)	1916 (57·0%)	0·99 (0·96–1·02)	0·99 (0·96–1·02)	0·99 (0·97–1·02)	1·00 (0·96–1·05)

Data are n (%), unless otherwise indicated. RR=relative risk.

* Adjusted for age.

† Adjusted for age, parity, neighbourhood income quintile, rural residence, stable and unstable chronic conditions, mental ill health, and substance use disorders.

‡ Adjusted for age, parity, neighbourhood income quintile, rural residence, stable and unstable chronic conditions, mental ill health, substance use disorders, smoking, overweight or obesity, prenatal care provider type, number of prenatal care visits, and prenatal class attendance.

## Data Availability

Data used for this study were housed at ICES, an independent not-forprofit corporation. The dataset used in this study is held securely in coded form at ICES. Although the data sharing agreements that govern the dataset prohibit making it publicly available, access might be granted to those who meet specified criteria for confidential access. External individuals must apply for access through ICES’ Data and Analytic Services (DAS), a division of ICES established to provide data and analytic services to third party researchers. The dataset that approved third party researchers would be permitted to access would be adjusted to ensure the risk of re-identification of any underlying individuals is low. Information about the application process, including the DAS Data Request Form and the criteria for access, including, for example, confirmation of approval by a research ethics board, are available at https://www.ices.on.ca/DAS/Submitting-your-request. For general information visit www.ices.on.ca/DAS or email das@ices.on.ca.

## Research in context

### Evidence before this study

We searched PubMed using the terms “disabilit*” and “breastfeed*”, for studies published in English, from database inception to March 31, 2022. We found three quantitative studies that examined breastfeeding rates in birthing parents with a disability, using data from a survey of the English National Health Service Trusts, the Rhode Island Pregnancy Risk Assessment Monitoring System, and the Massachusetts Pregnancy to Early Life Longitudinal database. Collectively, these studies showed lower rates of self-reported breastfeeding shortly after delivery in individuals with a disability compared with those without a disability. However, other WHO-UNICEF Baby Friendly Hospital Initiative breastfeeding indicators have not been examined in this population.

### Added value of this study

This study leverages health administrative data from ICES and the Better Outcomes Registry & Network to examine breastfeeding practices and supports during the birth hospital stay in all birthing parents with and without a disability in Ontario, Canada. To our knowledge, our study is the first to address several WHO-UNICEF Baby Friendly Hospital Initiative quality of care indicators in people with a disability, including the opportunity to initiate breastfeeding after birth, breastfeeding exclusively at discharge, and skin-to-skin contact—all of which predict breastfeeding exclusively at 6 months. To our knowledge, our study is also the first to report on gaps in receipt of professional breastfeeding supports after delivery in people with a disability. Finally, our study is one of the first cross-disability studies, examining people with physical, sensory, intellectual or developmental, and multiple disabilities separately compared with those without a disability. We found disparities in breastfeeding practices and supports in people with intellectual or developmental disabilities and multiple disabilities, compared with those without a disability, which were somewhat attenuated in sensitivity analyses that examined the roles of explanatory factors.

### Implications of all the available evidence

The absence of disparities for people with physical and sensory disabilities compared with those without disabilities was a positive finding of our study. However, our findings have implications for structural and individual supports to optimise breastfeeding outcomes for people with intellectual or developmental disabilities and multiple disabilities. Our data show the need for further research to better understand the factors that affect breastfeeding intentions, practices, and supports in these groups. Such research could ultimately inform training of health-care providers on the needs of birthing parents with intellectual or developmental disabilities or multiple disabilities and development of preconception and prenatal health promotion and education efforts to support informed decision making. These efforts should be undertaken in partnership with disability advocacy organisations to ensure they meet the needs of people with disabilities.
