# Supplementary material for: Disability and in-hospital breastfeeding practices and supports in Ontario, Canada: a population-based study
Source: Lancet Public Health. Author manuscript; Available in PMC 2023 Jan 10. (PMC9831273; doi:10.1016/S2468-2667(22)00310-3)
Supplement: 1 [file NIHMS1862585-supplement-1.pdf]

THE LANCET
Public Health

Supplementary appendix

This appendix formed part of the original submission and has been peer reviewed. We post it as supplied by the authors.

Supplement to: Brown HK, Taylor C, Vigod SN, et al. Disability and in-hospital breastfeeding practices and supports in Ontario, Canada: a population-based study. *Lancet Public Health* 2023; **8:** e47–56.

Figure S1. Derivation of the study cohort.

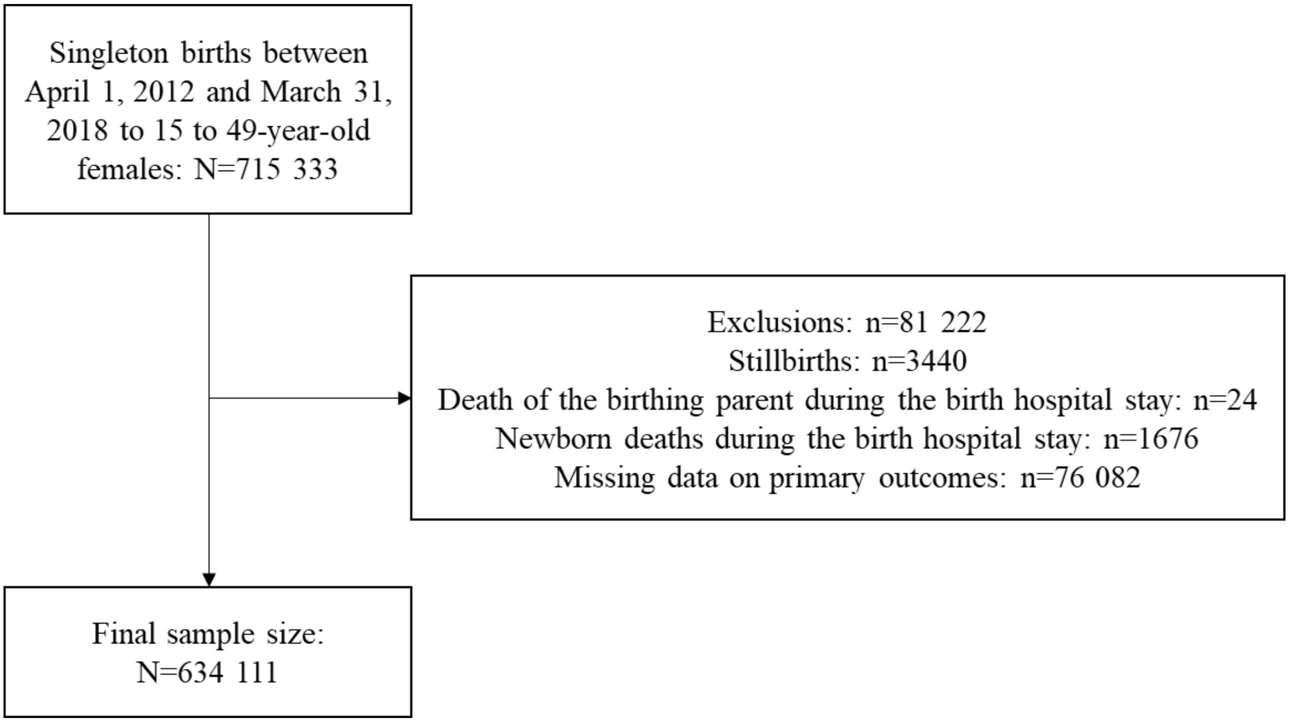

Table S1. Comparison of people who were included and not included in the cohort because of missing data on the primary outcomes.

Variable	Included in the cohort n=634 111	Not included in the cohort n=76 082	Standardized difference
Age, years			
15-24	22 218 (3·5)	1743 (2·3)	0.07
25-34	494 845 (78·0)	54 960 (72·2)	0.13
35-49	117 048 (18·5)	19 379 (25·5)	0.17
Multiparous	364 550 (57·5)	41 502 (54·5)	0.06
Neighbourhood income quintile (Q)			
Q1 (lowest)	140 309 (22·1)	14 051 (18·5)	0.09
Q2	127 459 (20·1)	14 683 (19·3)	0.02
Q3	129 733 (20·5)	16 015 (21·0)	0.01
Q4	131 829 (20·8)	16 787 (22·1)	0.03
Q5 (highest)	102 657 (16·2)	14 267 (18·8)	0.07
Missing	2124 (0·3)	279 (0·4)	0.01
Rural region of residence	29 773 (4·7)	1387 (1·8)	0.16
Stable chronic conditions	142 979 (22·5)	20 848 (27·4)	0.11
Unstable chronic conditions	80 456 (12·7)	11 563 (15·2)	0.07
Mental illness	237 819 (37·5)	28 620 (37·6)	0.00
Substance use disorder	26 613 (4·2)	2414 (3·2)	0.05
Prenatal smoking	69 235 (10·9)	4663 (6·1)	0.17
Pre-pregnancy overweight or obesity	250 732 (39·5)	23 363 (30·7)	0.19
Type of prenatal care provider			
Family physician	96 606 (15·2)	7935 (10·4)	0.14
Obstetrician	223 894 (35·3)	35 145 (46·2)	0.22
Shared care	260 497 (41·1)	31 743 (41·7)	0.01
Midwife	7073 (1·1)	145 (0·2)	0.12
None	46 041 (7·3)	1114 (1·5)	0.29
Number of prenatal care visits			
≤ 10	144 617 (22·8)	7662 (10·1)	0.35
11-14	171 795 (27·1)	19 500 (25·6)	0.03
≥ 15	317 699 (50·1)	48 920 (64·3)	0.29
Prenatal class attendance	142 879 (22·5)	15 929 (20·9)	0.04
Intention to breastfeed	565 051 (89·1)	51 581 (67·8)	0.54
Caesarean delivery	171 642 (27·1)	25 603 (33·7)	0.14
Severe maternal morbidity	12 439 (2·0)	1975 (2·6)	0.04
Preterm birth < 37 weeks	37 711 (5·9)	5869 (7·7)	0.07
NICU admission	73 617 (11·6)	10 011 (13·2)	0.05
Newborn discharge to social services	2499 (0·4)	255 (0·3)	0.01

Table S2. Conceptualization of study covariates.

Variable	Definition	Categories for analysis	Data source(s)
Age	Age at conception	15-24, 25-34, 35-49 years	RPDB
Parity	Number of prior livebirths or stillbirths	Primiparous, multiparous	MOMBABY
Neighbourhood income quintile	Dissemination area-level median income at conception	Q1 (lowest), Q2, Q3, Q4, Q5 (highest)	RPDB, linked with Census
Rural residence	Area of residence at conception, calculated based on sum of indicators related to travel time to nearest basic referral centre, travel time to nearest advanced referral centre, community population size, number of active general practitioners, population to general practitioner ratio, presence of a hospital, availability of ambulance services, social indicators (e.g., no airport, university, or community college), weather conditions (e.g., extreme rainfall, snowfall, or cold temperature), and selected services (e.g., anesthetic and obstetrical services)[25]	Rural, urban	RPDB
Chronic conditions	Johns Hopkins Adjusted Clinical Groups® System version 10 collapsed ambulatory diagnostic groups, classified as stable or unstable conditions in the 2 years before conception, the latter defined based on likelihood of complications and need for resources such as specialty care[26]	Present, absent (for both stable and unstable chronic conditions)	DAD, OHIP, OMHRS, NACRS
Mental illness	Psychotic, mood or anxiety, or other mental disorders in the 2 years before conception	Present, absent	DAD, OHIP, OMHRS, NACRS
Substance use disorder	Alcohol or illicit substance use disorder in the 2 years before conception	Present, absent	DAD, OHIP, OMHRS, NACRS
Smoking in pregnancy	Self-reported amount of smoking per day at time of first prenatal visit or birth hospital admission	Smoking, no smoking	BORN
Overweight/obesity	Maternal BMI, based on self-reported weight prior to pregnancy	Obese/overweight, not obese/overweight	BORN
Type of prenatal care provider	Health care provider delivering the majority of prenatal care (75%)	Family physician, obstetrician, midwife, shared care, none	BORN, OHIP
Number of prenatal care visits	Number of visits to a family physician, obstetrician, or midwife during pregnancy for antenatal care	≤ 10, 11-14, ≥ 15	BORN, OHIP
Prenatal class attendance	Attendance of any prenatal classes during pregnancy, including online education requiring registration or enrollment as well as in-person classes (not including prenatal education during routine antenatal care visits)	Present, absent	BORN
Intention to breastfeed	Parent intends to feed infant breastmilk, regardless of the method of feeding, as self-reported during pregnancy or at the time of birth	Intending, not intending	BORN
Caesarean section	Delivery via elective or emergency caesarean section	Caesarean, vaginal	MOMBABY
Severe maternal morbidity	Serious obstetric morbidity in pregnancy or during the birth hospital stay	Present, absent	DAD, MOMBABY
Preterm birth	Preterm birth at less than 37 weeks gestational age	Preterm, term	MOMBABY
NICU admission	Admission of the newborn to a neonatal intensive care unit during the birth hospital stay	Admitted, not admitted	MOMBABY
Infant discharge to social services	Infant discharged to social services (i.e., child welfare) after the birth hospital stay based on discharge location information	Discharged to social services, not discharged to social services	MOMBABY

Abbreviations: BORN = Better Outcomes Registry & Network; DAD = Discharge Abstract Database; OHIP = Ontario Health Insurance Plan database; OMHRS = Ontario Mental Health Reporting System; NACRS = National Ambulatory Care Reporting System; RPDB = Registered Persons Database.

Figure S2. Conceptualization of study variables.

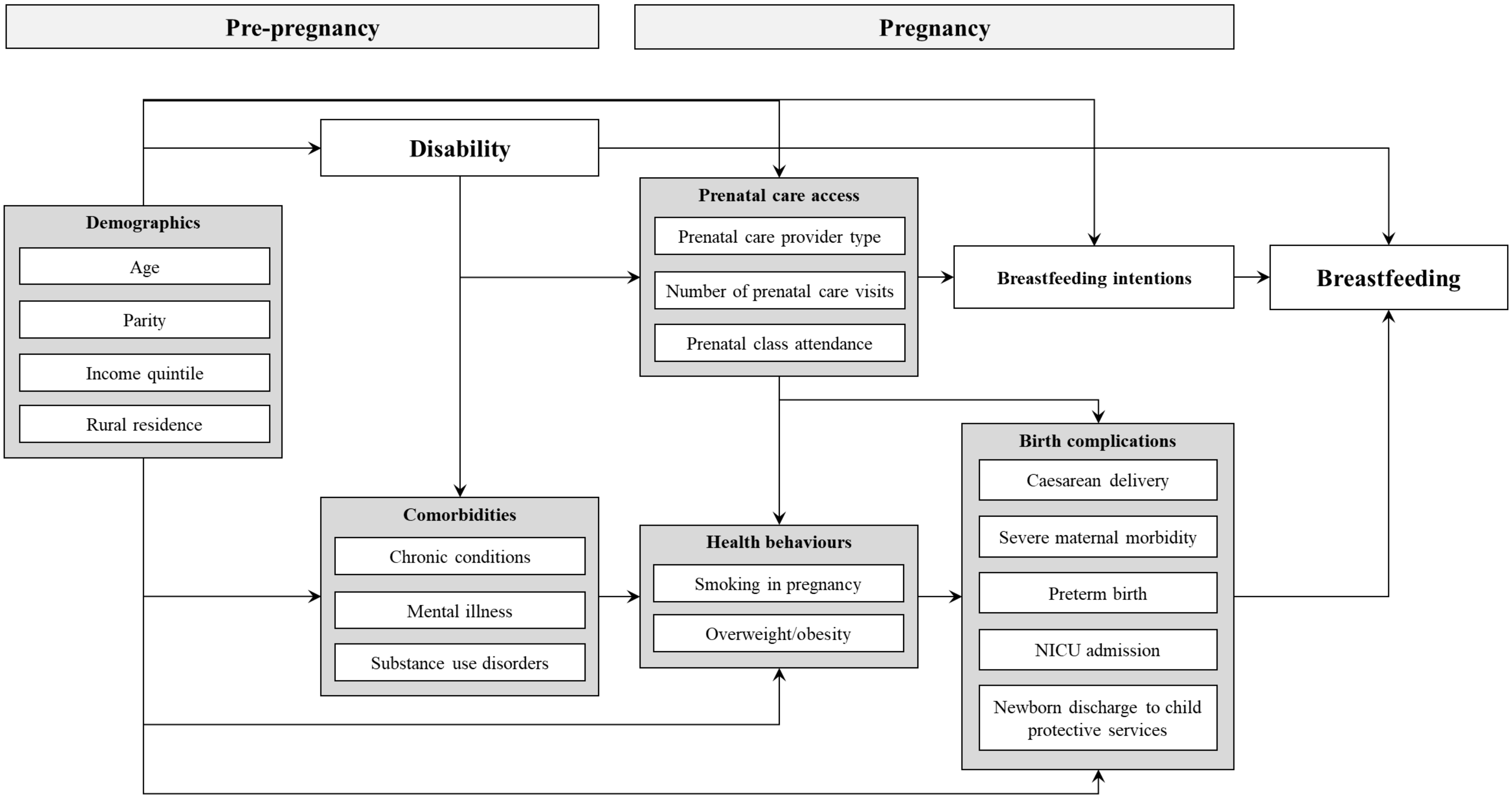

Table S3. Breastfeeding practices and receipt of supports that promote exclusive breastfeeding during the birth hospital stay, in people with a disability, compared to those without any recognized disability, by specific disability subtype.

Disability type	Number (%) with outcome	Unadjusted RR (95% CI)	Adjusted RR (95% CI)[a]	Adjusted RR (95% CI)[b]	Adjusted RR (95% CI)[c]
**Opportunity to latch within 2 hours of birth**					
No disability (n=555 310)	305 882 (55·1)	1·00 (Referent)	1·00 (Referent)	1·00 (Referent)	1·00 (Referent)
Physical - Musculoskeletal only (n=23 414)	12 912 (55·1)	1·00 (0·99-1·01)	1·00 (0·99-1·01)	1·00 (0·99-1·01)	1·01 (0·99-1·03)
Physical - Neurological only n=13 643)	7173 (52·6)	0·96 (0·94-0·97)	0·96 (0·94-0·98)	0·97 (0·96-0·99)	0·99 (0·96-1·01)
Physical - Permanent injury only (n=8594)	4705 (54·7)	0·99 (0·98-1·02)	1·00 (0·98-1·02)	1·01 (0·99-1·03)	1·01 (0·98-1·04)
Physical - Congenital anomalies only (n=4584)	2462 (53·7)	0·98 (0·95-1·00)	0·98 (0·96-1·01)	0·99 (0·96-1·02)	0·99 (0·95-1·03)
Physical - Multiple (n=4241)	2153 (50·8)	0·92 (0·89-0·95)	0·92 (0·89-0·95)	0·94 (0·91-0·97)	0·95 (0·91-1·00)
Sensory - Hearing loss only (n=13 869)	7604 (54·8)	1·00 (0·98-1·01)	1·00 (0·98-1·01)	1·00 (0·99-1·02)	1·01 (0·98-1·03)
Sensory - Vision only (n=5162)	2777 (53·8)	0·98 (0·95-1·00)	0·98 (0·95-1·00)	0·98 (0·96-1·01)	0·99 (0·95-1·02)
Sensory - Multiple (n=196)	118 (60·2)	1·09 (0·97-1·23)	1·09 (0·97-1·23)	1·10 (0·98-1·24)	1·10 (0·92-1·32)
IDD - Autism only (n=220)	121 (55·0)	1·00 (0·88-1·12)	1·02 (0·91-1·15)	1·06 (0·94-1·19)	1·07 (0·89-1·28)
IDD - Other IDD only (n=775)	306 (39·5)	0·72 (0·66-0·79)	0·73 (0·66-0·79)	0·76 (0·69-0·83)	0·77 (0·69-0·87)
IDD - Multiple (n=53)	20 (37·7)	0·67 (0·46-0·96)	0·70 (0·49-1·00)	0·76 (0·53-1·08)	0·81 (0·52-1·25)
Multiple - Physical + sensory (n=3516)	1814 (51·6)	0·94 (0·91-0·97)	0·94 (0·91-0·97)	0·96 (0·93-0·99)	0·97 (0·93-1·02)
Multiple - Physical + IDD (n=340)	130 (38·2)	0·69 (0·60-0·80)	0·69 (0·60-0·80)	0·73 (0·64-0·84)	0·76 (0·64-0·91)
Multiple - Sensory + IDD (n=117)	56 (47·9)	0·88 (0·72-1·08)	0·91 (0·75-1·11)	0·96 (0·79-1·16)	0·97 (0·75-1·27)
Multiple - All three types (n=77)	27 (35·1)	0·64 (0·47-0·88)	0·64 (0·47-0·88)	0·68 (0·50-0·92)	0·71 (0·48-1·03)
**Any breastfeeding before discharge**					
No disability (n=555 310)	482 702 (86·9)	1·00 (Referent)	1·00 (Referent)	1·00 (Referent)	1·00 (Referent)
Physical - Musculoskeletal only (n=23 414)	20 105 (85·9)	0·99 (0·98-0·99)	0·98 (0·98-0·99)	0·99 (0·98-0·99)	0·99 (0·98-1·01)
Physical - Neurological only n=13 643)	11 227 (82·3)	0·95 (0·94-0·96)	0·95 (0·94-0·96)	0·96 (0·95-0·97)	0·97 (0·95-0·99)
Physical - Permanent injury only (n=8594)	7240 (84·2)	0·97 (0·96-0·98)	0·98 (0·97-0·99)	0·99 (0·98-1·00)	1·00 (0·97-1·02)
Physical - Congenital anomalies only (n=4584)	3840 (83·8)	0·97 (0·95-0·98)	0·98 (0·96-0·99)	0·98 (0·97-0·99)	0·99 (0·95-1·02)
Physical - Multiple (n=4241)	3469 (81·8)	0·94 (0·92-0·95)	0·94 (0·93-0·95)	0·96 (0·94-0·97)	0·97 (0·94-1·00)
Sensory - Hearing loss only (n=13 869)	11 644 (84·0)	0·97 (0·96-0·97)	0·97 (0·96-0·98)	0·97 (0·97-0·98)	0·98 (0·96-1·00)
Sensory - Vision only (n=5162)	4465 (86·5)	0·99 (0·98-1·01)	0·99 (0·98-1·00)	0·99 (0·98-1·00)	0·99 (0·96-1·02)
Sensory - Multiple (n=196)	170 (86·7)	0·99 (0·94-1·05)	0·99 (0·93-1·05)	1·00 (0·94-1·06)	1·00 (0·86-1·16)
IDD - Autism only (n=220)	175 (79·5)	0·91 (0·84-0·98)	0·93 (0·86-1·00)	0·95 (0·89-1·03)	0·96 (0·83-1·12)
IDD - Other IDD only (n=775)	534 (68·9)	0·80 (0·76-0·84)	0·81 (0·77-0·85)	0·84 (0·80-0·88)	0·85 (0·78-0·93)
IDD - Multiple (n=53)	22 (41·5)	0·47 (0·34-0·66)	0·50 (0·36-0·68)	0·53 (0·38-0·73)	0·56 (0·37-0·85)
Multiple - Physical + sensory (n=3516)	2858 (81·3)	0·94 (0·92-0·96)	0·94 (0·93-0·96)	0·96 (0·94-0·97)	0·96 (0·93-1·00)
Multiple - Physical + IDD (n=340)	219 (64·4)	0·74 (0·68-0·81)	0·75 (0·69-0·82)	0·78 (0·72-0·85)	0·81 (0·71-0·92)
Multiple - Sensory + IDD (n=117)	74 (63·2)	0·74 (0·64-0·86)	0·75 (0·65-0·87)	0·78 (0·67-0·90)	0·79 (0·63-1·00)
Multiple - All three types (n=77)	36 (46·8)	0·58 (0·45-0·74)	0·58 (0·46-0·75)	0·61 (0·48-0·78)	0·63 (0·45-0·87)
**Exclusive breastfeeding before discharge**					
No disability (n=555 310)	327 981 (59·1)	1·00 (Referent)	1·00 (Referent)	1·00 (Referent)	1·00 (Referent)
Physical - Musculoskeletal only (n=23 414)	13 868 (59·2)	1·00 (0·99-1·01)	1·00 (0·99-1·01)	1·01 (1·00-1·02)	1·03 (1·01-1·04)

Disability type	Number (%) with outcome	Unadjusted RR (95% CI)	Adjusted RR (95% CI)[a]	Adjusted RR (95% CI)[b]	Adjusted RR (95% CI)[c]
Physical - Neurological only n=13 643)	7579 (55·6)	0·95 (0·93-0·96)	0·95 (0·93-0·96)	0·97 (0·95-0·98)	0·99 (0·97-1·01)
Physical - Permanent injury only (n=8594)	5165 (60·1)	1·02 (1·00-1·04)	1·02 (1·00-1·04)	1·03 (1·01-1·05)	1·04 (1·01-1·06)
Physical - Congenital anomalies only (n=4584)	2675 (58·4)	0·99 (0·97-1·02)	0·99 (0·97-1·02)	1·00 (0·97-1·02)	1·00 (0·96-1·04)
Physical - Multiple (n=4241)	2309 (54·4)	0·92 (0·89-0·95)	0·92 (0·90-0·95)	0·95 (0·92-0·98)	0·98 (0·94-1·03)
Sensory - Hearing loss only (n=13 869)	8132 (58·6)	0·99 (0·98-1·01)	0·99 (0·98-1·01)	1·00 (0·98-1·02)	1·00 (0·98-1·03)
Sensory - Vision only (n=5162)	2900 (56·2)	0·95 (0·92-0·97)	0·95 (0·93-0·98)	0·96 (0·94-0·99)	0·97 (0·94-1·01)
Sensory - Multiple (n=196)	114 (58·2)	1·00 (0·88-1·13)	0·99 (0·88-1·12)	1·01 (0·89-1·14)	1·01 (0·84-1·22)
IDD - Autism only (n=220)	94 (42·7)	0·71 (0·61-0·84)	0·71 (0·61-0·84)	0·75 (0·64-0·88)	0·77 (0·63-0·95)
IDD - Other IDD only (n=775)	310 (40·0)	0·70 (0·64-0·76)	0·70 (0·64-0·76)	0·74 (0·68-0·81)	0·76 (0·68-0·85)
IDD - Multiple (n=53)	10 (18·9)	0·35 (0·20-0·61)	0·35 (0·20-0·62)	0·40 (0·23-0·70)	0·43 (0·23-0·80)
Multiple - Physical + sensory (n=3516)	1877 (53·4)	0·91 (0·88-0·94)	0·91 (0·88-0·94)	0·94 (0·91-0·97)	0·96 (0·92-1·01)
Multiple - Physical + IDD (n=340)	128 (37·6)	0·64 (0·55-0·74)	0·64 (0·55-0·74)	0·69 (0·59-0·80)	0·73 (0·61-0·87)
Multiple - Sensory + IDD (n=117)	42 (35·9)	0·64 (0·50-0·82)	0·64 (0·49-0·82)	0·68 (0·53-0·88)	0·69 (0·51-0·94)
Multiple - All three types (n=77)	18 (23·4)	0·43 (0·29-0·64)	0·43 (0·28-0·64)	0·46 (0·31-0·68)	0·48 (0·30-0·77)
**Skin-to-skin contact with the birthing parent within 2 hours of birth**					
No disability (n=555 310)	430 762 (77·6)	1·00 (Referent)	1·00 (Referent)	1·00 (Referent)	1·00 (Referent)
Physical - Musculoskeletal only (n=23 414)	18 053 (77·1)	0·99 (0·99-1·00)	1·00 (0·99-1·00)	1·00 (0·99-1·01)	1·00 (0·99-1·02)
Physical - Neurological only n=13 643)	10 233 (75·0)	0·97 (0·96-0·98)	0·97 (0·96-0·98)	0·98 (0·97-0·99)	0·98 (0·96-1·00)
Physical - Permanent injury only (n=8594)	6684 (77·8)	1·00 (0·99-1·02)	1·00 (0·99-1·02)	1·01 (0·99-1·02)	1·01 (0·98-1·03)
Physical - Congenital anomalies only (n=4584)	3489 (76·1)	0·98 (0·96-1·00)	0·98 (0·96-0·99)	0·98 (0·96-0·99)	0·98 (0·95-1·01)
Physical - Multiple (n=4241)	3087 (72·8)	0·94 (0·92-0·96)	0·94 (0·92-0·96)	0·95 (0·93-0·97)	0·96 (0·92-0·99)
Sensory - Hearing loss only (n=13 869)	10 586 (76·3)	0·98 (0·97-0·99)	0·98 (0·97-0·99)	0·98 (0·97-0·99)	0·99 (0·97-1·01)
Sensory - Vision only (n=5162)	3870 (75·0)	0·97 (0·95-0·98)	0·97 (0·95-0·98)	0·97 (0·96-0·99)	0·97 (0·94-1·01)
Sensory - Multiple (n=196)	144 (73·5)	0·95 (0·87-1·03)	0·94 (0·86-1·03)	0·95 (0·87-1·03)	0·95 (0·80-1·12)
IDD - Autism only (n=220)	159 (72·3)	0·93 (0·86-1·01)	0·94 (0·86-1·02)	0·95 (0·88-1·03)	0·95 (0·81-1·11)
IDD - Other IDD only (n=775)	534 (68·9)	0·89 (0·85-0·93)	0·89 (0·84-0·93)	0·90 (0·86-0·95)	0·91 (0·83-0·99)
IDD - Multiple (n=53)	27 (50·9)	0·65 (0·50-0·85)	0·64 (0·49-0·85)	0·67 (0·51-0·88)	0·69 (0·47-1·01)
Multiple - Physical + sensory (n=3516)	2560 (72·8)	0·94 (0·92-0·96)	0·94 (0·92-0·96)	0·95 (0·93-0·97)	0·96 (0·92-0·99)
Multiple - Physical + IDD (n=340)	213 (62·6)	0·81 (0·74-0·88)	0·80 (0·73-0·87)	0·82 (0·75-0·89)	0·83 (0·72-0·95)
Multiple - Sensory + IDD (n=117)	72 (61·5)	0·80 (0·69-0·93)	0·82 (0·71-0·94)	0·83 (0·72-0·96)	0·84 (0·67-1·06)
Multiple - All three types (n=77)	41 (53·2)	0·70 (0·56-0·86)	0·69 (0·56-0·86)	0·71 (0·57-0·88)	0·72 (0·53-0·98)
**Provision of assistance with breastfeeding within 6 hours of birth after initial feeding**					
No disability (n=555 310)	297 278 (53·5)	1·00 (Referent)	1·00 (Referent)	1·00 (Referent)	1·00 (Referent)
Physical - Musculoskeletal only (n=23 414)	12 512 (53·4)	1·00 (0·99-1·01)	1·00 (0·99-1·01)	1·00 (0·99-1·02)	1·01 (0·99-1·03)
Physical - Neurological only n=13 643)	6914 (50·7)	0·96 (0·94-0·97)	0·96 (0·94-0·97)	0·97 (0·95-0·98)	0·98 (0·96-1·00)
Physical - Permanent injury only (n=8594)	4704 (54·7)	1·02 (1·00-1·04)	1·02 (1·00-1·04)	1·02 (1·01-1·04)	1·03 (1·00-1·06)
Physical - Congenital anomalies only (n=4584)	2387 (52·1)	0·97 (0·95-1·00)	0·97 (0·95-1·00)	0·98 (0·95-1·00)	0·98 (0·94-1·02)
Physical - Multiple (n=4241)	2155 (50·8)	0·96 (0·93-0·99)	0·96 (0·93-0·99)	0·97 (0·94-1·00)	0·99 (0·95-1·03)
Sensory - Hearing loss only (n=13 869)	7354 (53·0)	0·99 (0·98-1·01)	0·99 (0·98-1·01)	1·00 (0·98-1·01)	1·00 (0·98-1·02)

Disability type	Number (%) with outcome	Unadjusted RR (95% CI)	Adjusted RR (95% CI)[a]	Adjusted RR (95% CI)[b]	Adjusted RR (95% CI)[c]
Sensory - Vision only (n=5162)	2735 (53·0)	0·99 (0·97-1·02)	0·99 (0·97-1·02)	0·99 (0·97-1·02)	1·00 (0·96-1·04)
Sensory - Multiple (n=196)	107 (54·6)	0·96 (0·85-1·09)	0·95 (0·83-1·08)	0·96 (0·84-1·09)	0·96 (0·79-1·16)
IDD - Autism only (n=220)	109 (49·5)	0·92 (0·80-1·04)	0·92 (0·81-1·05)	0·95 (0·84-1·08)	0·96 (0·79-1·16)
IDD - Other IDD only (n=775)	334 (43·1)	0·82 (0·75-0·89)	0·81 (0·75-0·88)	0·84 (0·78-0·91)	0·86 (0·77-0·96)
IDD - Multiple (n=53)	14 (26·4)	0·49 (0·31-0·78)	0·51 (0·33-0·80)	0·55 (0·35-0·86)	0·57 (0·34-0·96)
Multiple - Physical + sensory (n=3516)	1802 (51·3)	0·97 (0·94-1·00)	0·97 (0·94-1·00)	0·98 (0·95-1·01)	0·99 (0·95-1·04)
Multiple - Physical + IDD (n=340)	124 (36·5)	0·70 (0·61-0·81)	0·72 (0·62-0·82)	0·75 (0·65-0·85)	0·77 (0·65-0·92)
Multiple - Sensory + IDD (n=117)	45 (38·5)	0·72 (0·56-0·91)	0·72 (0·57-0·92)	0·75 (0·59-0·95)	0·76 (0·56-1·02)
Multiple - All three types (n=77)	25 (32·5)	0·61 (0·44-0·86)	0·61 (0·44-0·86)	0·64 (0·46-0·89)	0·66 (0·45-0·98)

[a] Adjusted for age.

[b] Adjusted for age, parity, neighbourhood income quintile, rural residence, stable and unstable chronic conditions, mental illness, and substance use disorders.

[c] Adjusted for age, parity, neighbourhood income quintile, rural residence, stable and unstable chronic conditions, mental illness, substance use disorders, smoking, overweight/obesity, prenatal care provider type, number of prenatal care visits, and prenatal class attendance.

Table S4. Reasons for giving fluids other than breastmilk, in people with a disability, compared to those without any recognized disability.[a]

Variable [b]	Physical disability only	Sensory disability only	Intellectual/ developmental disability only	Multiple disabilities	No disability
	n=17 698	n=6191	n=437	n=1455	n=178 772
Maternal medical	407 (2·3)	111 (1·8)	7 (1·6)	45 (3·1) [a]	2738 (1·5)
Infant medical	2546 (14·4)	962 (15·5)	41 (9·4) [a]	205 (14·1)	25 252 (14·1)
Informed parent decision	15 375 (86·9)	5359 (86·6)	377 (86·3)	1250 (85·9)	157 171 (87·9)
Birth mother not involved in care	97 (0·5)	37 (0·6)	24 (5·5) [a]	24 (1·6) [a]	481 (0·3)

[a] Analysis restricted to newborns receiving fluids other than breastmilk.
[b] Standardized difference > 0·10, comparing people within each respective disability group to those without a disability.
